# Economic Evaluation of Comprehensive Genomic Profiling in an Advanced Solid Cancer Population

**DOI:** 10.1001/jamanetworkopen.2025.48538

**Published:** 2025-12-11

**Authors:** Lucas F. van Schaik, Brigitte Maes, Pieter-Jan Volders, Guy Froyen, Philippe Aftimos, Evandro de Azambuja, Hedwig M. Blommestein, Wim H. van Harten, Valesca P. Retèl

**Affiliations:** 1 Division of Psychosocial Research and Epidemiology, Netherlands Cancer Institute, Amsterdam, the Netherlands; 2 Laboratory for Molecular Diagnostics, Department of Clinical Biology, Jessa Hospital, Hasselt, Belgium; 3 Faculty of Medicine and Life Sciences, Limburg Clinical Research Centre, University of Hasselt, Hasselt, Belgium; 4 Erasmus School of Health Policy and Management, Erasmus University Rotterdam, Rotterdam, the Netherlands; 5 Hôpital Universitaire de Bruxelles, Université Libre de Bruxelles, Institut Jules Bordet, Brussels, Belgium; 6 Department of Health Technology and Services Research, University of Twente, Enschede, the Netherlands

## Abstract

**Importance:**

Developments in genomic profiling introduced larger gene panels with decreasing sequencing costs. Although the feasibility of comprehensive genomic profiling (CGP) has been demonstrated, its health economic impact remains uncertain.

**Objective:**

To provide insights into the costs related to clinical implementation of CGP-based advice in a large prospective cohort.

**Design, Setting, and Participants:**

In this economic evaluation of a prospective Belgian multicenter observational study (Belgian Approach of Local Laboratory Extensive Tumor Testing [BALLETT]), a decision tree was designed to illustrate costs and outcomes adopting a diagnostic time horizon. The evaluation also included a future evaluation scenario comparing standard-of-care diagnostics with upfront CGP. This study included patients with advanced solid tumors enrolled in the BALLETT study between May 1, 2021, and October 31, 2023. Analyses were conducted using the entire study population and individual tumor types.

**Exposure:**

CGP followed by molecular tumor board (MTB) discussions to formulate treatment recommendations.

**Main Outcomes and Measures:**

Model outcomes were total diagnostic costs and the percentage of patients with actionable targets, MTB recommendations, and matched treatments. Incremental cost-consequence ratios were calculated.

**Results:**

A total of 814 patients with advanced tumors (mean [SD] age, 60.8 [12.3] years; 452 female [55.5%]) participated in the study. The mean diagnostic costs for patients of the BALLETT cohort were €2147. Actionable targets were identified in 621 patients (76%), MTB recommendations were provided in 529 patients (65%), and 123 patients (15%) received a matched treatment. The diagnostic cost to match a CGP-matched treatment was €14 249. This cost varied between specific tumor types (€9952 for lung cancer to €20 377 for colon cancer). Sensitivity analyses showed that these cost-consequence ratios are most influenced by diagnostic cost and the uptake of MTB recommendations. In the scenario comparing upfront CGP and standard-of-care diagnostics, the incremental diagnostic cost to match a treatment was estimated to be slightly lower (€13 936).

**Conclusions and Relevance:**

This economic evaluation of CGP in patients with advanced cancer estimated the diagnostic cost to match a treatment. CGP-matched treatment cost and benefit requirements were estimated under which CGP was cost-effective, thereby providing health care decision-makers with relevant economic evidence for decision-making.

## Key Points

**Question:**

What are the health economic consequences of comprehensive genomic profiling (CGP) in an advanced pancancer population?

**Findings:**

This economic evaluation with 814 participants found that diagnostic costs amount to €2816 to identify 1 patient with an actionable target to €14 249 to identify 1 patient receiving a CGP-matched treatment. Adopting a conceptual willingness-to-pay threshold of €5000 per matched treatment, a CGP cost reduction to €1250 combined with an increased uptake of molecular tumor board recommendations to 47% was required to achieve a positive net monetary benefit.

**Meaning:**

These results suggest that substantial diagnostic costs made to match 1 CGP-informed treatment can be reduced most effectively by lowering diagnostic costs and increasing uptake of molecular tumor board recommendations.

## Teaser

This economic evaluation of patients with advanced cancer addresses some of the challenges of conducting a full cost-effectiveness analysis of comprehensive genomic profiling and provides insights into the associated benefits and costs of comprehensive genomic profiling alongside the diagnostic pathway.

## Introduction

Molecular profiling plays a critical role in modern oncology by providing genomic insights that assist and inform tumor diagnosis, prognosis, or treatment decisions.^1^ Targets can be identified with single-gene tests, next-generation sequencing (NGS) panels with a limited number of genes, or comprehensive genomic profiling (CGP). CGP consists of large NGS panels or even exome or genome sequencing and identifies many targets simultaneously, such as copy number alterations or fusions, which may be especially important when many targeted treatments are available.^2^ CGP also uncovers targets beyond standard of care (SOC) for early-access, off-label, or investigational treatment options.^3^ Although the benefits of such treatments are uncertain, the literature suggests that biomarker-based matching of treatments may be associated with improved response rates and progression-free survival.^4,5,6,7^ The Belgian BALLETT (Belgian Approach of Local Laboratory Extensive Tumor Testing) study demonstrated the feasibility of decentralized CGP in patients with advanced cancer. Actionable targets were identified in 81% of patients with successful CGP, resulting in molecular tumor board (MTB) treatment recommendations in 69% of them. Eventually, 23% of these MTB recommendations led to matched treatments.^8^

Economic evaluations, initiated to support decision-makers to allocate resources efficiently, calculate costs and effects of alternative strategies. Preferably, economic evaluations involving single-gene tests or NGS include both costs and effects of tests itself and of subsequent treatments assigned after test results. Many economic analyses are performed for single-gene tests or NGS in the setting of one tumor type or one target.^9^ However, because CGP identifies various targets for treatment options that might not have been considered before, it disrupts this traditional companion test-treatment relation. Economic evaluations for CGP are further complicated by CGP’s potential range of benefits, pancancer application, and lack of comparative studies including survival outcomes.^3,10^

The few available economic analyses of CGP often relied on theoretical diagnostic pathways to assess CGP’s economic impact without incorporating empirical data of patients receiving CGP.^9,11,12,13,14^ Although providing valuable insights, they do not reflect actual treatment decisions after CGP in clinical practice. Using single-arm, nationwide BALLETT study data, we conducted a pragmatic economic evaluation that combined elements of cost-consequence and cost-effectiveness analyses. This approach was adopted to address some of the challenges of conducting a full cost-effectiveness analysis of CGP and to provide insights into the associated benefits and costs of CGP alongside the diagnostic pathway.

## Methods

### BALLETT Study

**Figure 1.**
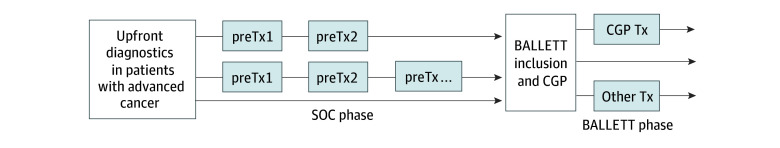
Patient Trajectory and Positioning of BALLETT Study Patients in the BALLETT study represent a pancancer solid tumor metastasized cohort with varying degrees of pretreatment (preTx). CGP Tx indicates comprehensive genomic profiling–informed matched treatment; other Tx, any other treatment that was not informed by comprehensive genomic profiling but received after BALLETT inclusion; and SOC, standard of care.

The BALLETT study evaluated the feasibility and clinical value of CGP. A total of 814 patients with advanced solid tumors, a life expectancy greater than 12 weeks, and varying degrees of pretreatment were prospectively included between May 1, 2021, and October 31, 2023 (Figure 1). CGP was provided in 9 laboratories with a commercial kit with high analytical sensitivity and specificity (>99%) (TruSight Oncology 500 kit, TSO500, Illumina). Variants were assessed by the Association for Molecular Pathology, American Society of Clinical Oncology, and College of American Pathologists tiering system: tier 1A/1B for strong clinical importance, and tier 2C/2D for potential clinical importance.^16^ Patients were discussed in the interdisciplinary national MTB to formulate MTB recommendations for matched treatments and further germline testing or counseling if necessary. Data were collected on CGP results, recommended treatments, and received treatments.^8^ Reporting followed the Consolidated Health Economic Evaluation Reporting Standards (CHEERS) reporting guideline.^15^ Written informed consent for BALLETT study participation was obtained by the Belgian study. This informed consent allowed the use of study data for research purposes and sharing with third parties for this goal. The institutional review board of the Antoni van Leeuwenhoek approved the economic analysis as described in this article.

### Model Description

**Figure 2.**
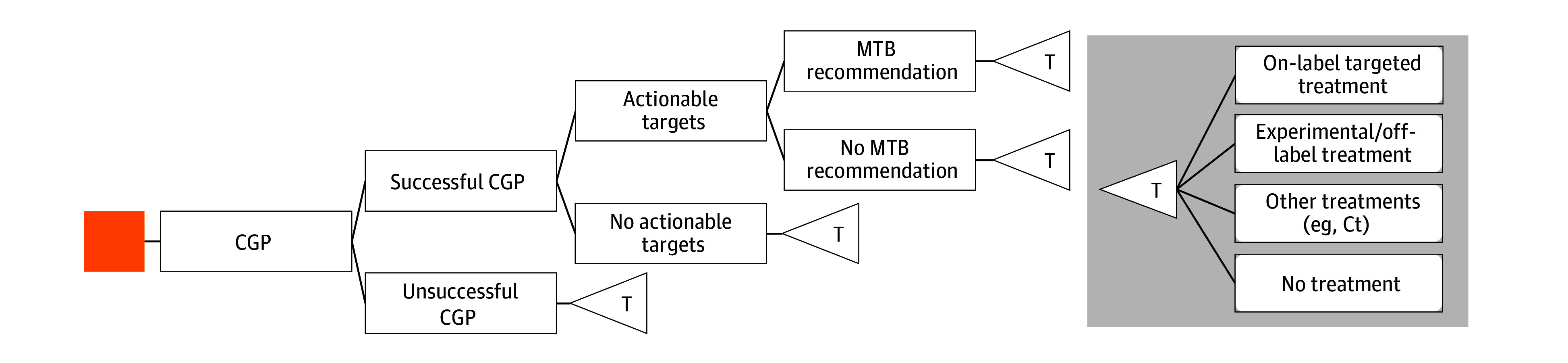
Decision Tree of Diagnostic Pathway of Comprehensive Genomic Profiling (CGP) The decision tree follows the sequential steps in the diagnostic pathway of the BALLETT study up to treatment allocation. The on-label and experimental or off-label treatment categories are both CGP-informed matched treatments, whereas other treatments and no treatment were not CGP informed. Ct indicates chemotherapy; MTB, molecular tumor board; T, treatment advice followed.

A decision tree was developed to illustrate the diagnostic pathway of the BALLETT study cohort to summarize costs and diagnostic outcomes of CGP in the Belgium setting from a health care perspective (Figure 2). Patients entered the decision tree when CGP was conducted. As the BALLETT study was single arm and did not capture survival data, we focused on the diagnostic setting and direct consequences of testing only. Therefore, our chosen diagnostic time horizon covers the period during diagnostic testing and allocation of subsequent treatments. In the final node of the decision tree, patients were categorized into 4 mutually exclusive treatment categories: (1) patients receiving matched approved on-label targeted treatment or on-label immunotherapy based on molecular findings; (2) patients receiving investigational or off-label targeted treatment based on a molecular findings; (3) patients receiving non–molecular-informed treatment types, such as non–biomarker-based immunotherapy, chemotherapy, or hormonal treatments; and (4) patients not receiving any treatment due to a variety of reasons.

Input probabilities for each step of the decision tree were informed by patient-level analysis of BALLETT study data. eTables 1 and 2 in Supplement 1 summarize each parameter and how it was derived. Treatments allocated after CGP were recorded in the BALLETT study and used to estimate probabilities for different treatment categories. eMethods 1 in Supplement 1 presents the steps taken to validate the decision tree (Advishe checklist^17^).

### Costs

CGP costs (€1831.94 [US $2106]) were obtained from a microcosting study that was conducted alongside the BALLETT study^19^ using the activity-based costing method of Pasmans et al.^18^ Repeated test costs were included by multiplying CGP costs with the probability of repeat CGP analysis obtained from BALLETT study data. Direct salary costs for medical specialists for MTB participation were estimated at €209.50 (US $241) per patient (eTable 3 in Supplement 1). Treatment costs were not included because a diagnostic time horizon was chosen.

### Statistical Analysis 

#### Model Output

The decision tree was used to summarize outcomes of interest for the entire BALLETT study cohort and separately for 4 individual tumor types within the cohort: sarcoma, lung, breast, and colon cancer. Model outcomes of interest were (1) diagnostic costs, (2) percentage of patients with actionable targets (at least 1 tier 1A/B or 2C/D variant), (3) percentage of patients who received MTB recommendations, and (4) percentage of patients who received a matched MTB-recommended treatment. Incremental cost-consequence ratios (ICCRs) were calculated using total diagnostic costs and estimated diagnostic outcomes, reflecting additional costs required to obtain 1 additional outcome unit. No discounting was included due to the diagnostic time horizon. All analyses were conducted using the DARTH framework in R software, version 2023.09 + 494 (R Foundation for Statistical Computing).^20^

#### Sensitivity Analyses

To conduct deterministic sensitivity analyses, we calculated the net monetary benefit (NMB) using a conceptual willingness-to-pay (WTP) threshold for the outcome-matched treatments. Typically, WTP thresholds are established for quality-adjusted life-years (QALYs), including both length and quality of life. Because we focused on matched treatments, we selected a hypothetical societally acceptable cost to match 1 treatment of €5000 (US $5750), representing approximately 4 times the highest reimbursement rate for SOC molecular diagnostics (€1200 [US $1380]).^21^ A positive NMB indicates that the monetized benefit (€5000 per matched treatment) outweighs associated diagnostic cost. Uncertainty in the estimated costs and outcomes was evaluated by a probabilistic sensitivity analysis simulating 1000 runs. β, Dirichlet, and γ distributions were derived for clinical and costs parameters from BALLETT study data, assuming a 10% SE in case of limited data.

#### Exploratory Long-Term Threshold Analysis

When deciding on reimbursement, health care decision-makers often assess the criterion cost-effectiveness, comparing incremental costs and effects (incremental cost-effectiveness ratio [ICER]) of a technology compared with current SOC. To establish when CGP would be cost-effective, an exploratory long-term threshold analysis was conducted. Because most patients had completed SOC diagnostics before enrollment in the BALLETT study, indicated by the principal investigator in discussions, we assumed a last-resort CGP setting for this analysis. As comparator, we assumed that without CGP, no further diagnostics would have been conducted, in line with completion of SOC diagnostics for most patients. Therefore, no diagnostic costs were attributed to this comparator. Furthermore, we assumed that treatment costs and benefits of patients without CGP-matched treatments were equal for the CGP and no diagnostics strategies, meaning that they have a zero net effect on the ICER calculation. Finally, we estimated the ICER while attributing varying incremental costs (∆costs) and QALYs (∆QALYs) to patients with CGP-matched treatments, relative to treatment costs and QALYs that these patients would have had under no diagnostics: ICER = (Costs CGP diagnostics + ΔCosts CGP Matched Treatments)/(ΔQALYs CGP Matched Treatments). The commonly used WTP threshold in Belgium of €40 000 (US $100 000 or US $150 000) per QALY was applied.^22,23^

#### Upfront Diagnostics Scenario

Beyond the last-resort setting, CGP also could be implemented as upfront companion diagnostic, where it may inform sequential treatment lines. Although not the context of the BALLETT study, by combining BALLETT study data with patient-level retrospective treatment data, a scenario analysis was developed comparing diagnostic costs and outcomes of upfront CGP with SOC diagnostics to illustrate upfront CGP’s potential diagnostic impact (Figure 1). The probability of actionable targets by upfront CGP was informed by BALLETT study data. For SOC diagnostics, actionable targets were obtained by filtering CGP findings, only keeping targets included in SOC diagnostic guidelines.^24^ The probability of matching a treatment for CGP was based on matched treatments provided during the BALLETT study and SOC phase, informed by retrospective treatment data covering the time before enrollment. For SOC diagnostics, this probability consisted only of matched treatments provided during the SOC phase before BALLETT enrollment. Weighted mean diagnostic costs for SOC diagnostics were estimated based on diagnostic guidelines and cost estimations of NGS panels (mean diagnostic cost, €403 [US $463]).^19^ Patients in whom CGP informed successive treatments (first on-label followed by investigational matched treatment) contributed twice to the outcome matched treatments. This analysis was conducted for the entire BALLETT population and for a subgroup only considering patients with a current indication for upfront SOC diagnostics because this was not true for all patients. eMethods 2 in Supplement 1 lists more details on this analysis.

## Results

### Base-Case Model Output

A total of 814 patients with advanced tumors (mean [SD] age, 60.8 [12.3] years; 452 [55.5%] female and 362 [44.5%] male) participated in the study. Using the decision tree to summarize outcomes for the BALLETT cohort, CGP resulted in at least one actionable target in 621 patients (76%), MTB recommendations in 529 patients (65%), and an MTB-recommended matched treatment in 123 patients (15%), at a mean diagnostic cost of €2147 (US $2469) (Table). Although the cost to identify a patient with an actionable target was €2816 (US $3238) (ICCR), the ICCR to identify a patient with a CGP-matched treatment increased to €14 249 (US $16 386). Mean diagnostic costs remained relatively stable among the tumor types, whereas benefits varied. Patients with actionable targets were relatively most present in breast (105 [88%]) and lung cancer (66 [87%]). Matched treatments were seen most in lung cancer (16 [21%]) and less in colon cancer (9 [10%]). Hence, the ICCR to match a targeted treatment was €9952 (US $11 445) for lung cancer, whereas it was more than 2 times higher for colon cancer (€20 377 [US $23 434]). The predefined WTP threshold of €5000 per matched treatment was exceeded in all base-case analyses.

**Table.** Base-Case and Scenario Analysis Results^a^

Variable	No. of patients	Diagnostic costs, €		Actionable targets, % (ICCR, €)	MTB recommendations, % (ICCR, €)	Total matched treatments, % (ICCR, €)	On-label matched treatments, %
		Mean diagnostic cost	Total diagnostic cost, €				
Base-case analysis CGP							
All tumor types	814	2147	1 747 684	76 (2816)	65 (3302)	15 (14 249)	4
Lung cancer	76	2095	159 232	87 (2413)	82 (2568)	21 (9952)	9
Breast cancer	120	2144	257 221	88 (2450)	73 (2923)	18 (11 846)	7
Colon cancer	86	2089	179 647	76 (2764)	63 (3327)	10 (20 377)	0
Sarcoma	53	2273	120 445	55 (4153)	40 (5735)	15 (15 568)	0
Scenario analysis							
Upfront SOC	814	429	349 235	37	NA	26	26
Upfront CGP	814	2152	1 751 943	81 (3925)	66 (NA)^b^	38 (13 936)^c^	27
Upfront SOC with indication for SOC diagnostics	422	828	349 235	51	NA	32	32
Upfront CGP with indication for SOC diagnostics	422	2157	910 307	85 (3857)	74 (NA)^b^	44 (10 483)^c^	33

Abbreviations: CGP, comprehensive genomic profiling; ICCR, incremental cost-consequence ratio; MTB, molecular tumor board; NA, not applicable; SOC, standard of care diagnostics.

^a^ Total costs and percentage of patients with clinical outcomes of interest for the base-case and scenario analysis. Actionable targets were percentage of patients with at least one tier 1A or 1B or 2C or 2D classified actionable target identified. MTB recommendations were percentage of patients with at least one recommendation formulated in the national MTB, translating actionable targets into actionable clinical decisions. Matched treatments were percentage of patients who actually received a matched treatment following the proposed MTB recommendation. To convert to euros to US dollars, multiply by 1.15.

^b^ No ICCR calculated as this outcome was not estimated for the SOC strategy.

^c^ In the scenario analysis, patients who received both an on-label matched treatment and an investigational or off-label matched treatment were counted twice toward the percentage of matched treatments outcome.

### Sensitivity Analyses

The NMB, using a WTP threshold of €5000 (US $5750) per matched treatment, was strongly influenced by uptake of MTB recommendations toward matched on-label or investigational treatments and CGP cost (eFigures 1 and 2 in Supplement 1). A positive NMB was only achieved at an uptake of MTB recommendations to any type of CGP-matched treatments of at least 66% (currently 23%) or a reduction of CGP cost to €507 (US $583) (currently €1832 [US $2107]). The combined effects of varying CGP cost reductions and increased uptake of MTB recommendations were shown in a 2-way sensitivity analysis, indicating a positive NMB at CGP costs of €1250 (US $1438) and MTB recommendation uptake to any treatment of 47%.

**Figure 3.**
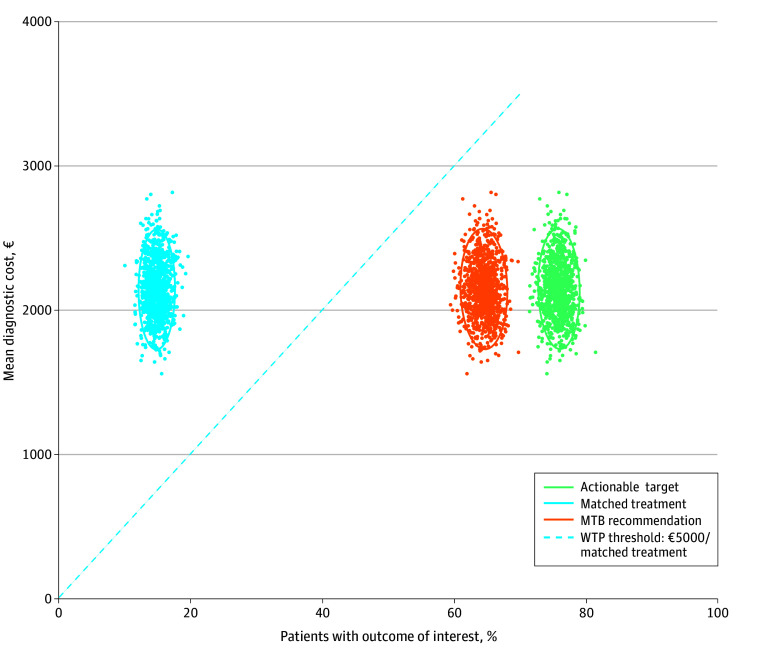
Cost-Consequence Plane Showing Costs and Percentage of Patients With Diagnostic Outcomes This plane shows the diagnostic costs and outcomes of 1000 simulations of the BALLETT cohort, with ellipses representing 95% CIs of the iterations. The figure shows the percentage of patients with actionable targets, molecular tumor board (MTB) recommendations, and matched treatments, plotted against the mean diagnostic costs. The dotted line in blue reflects the hypothetical willingness-to-pay (WTP) threshold of €5000 per matched treatment, which is exceeded in all 1000 simulations.

Input parameters were sampled from estimated distributions for the probabilistic sensitivity analysis (eFigure 3 in Supplement 1). The probabilistic sensitivity analysis showed that the uncertainty was mainly seen in diagnostic costs (95% CI, €1793-2556 [US $2061-2939]), whereas estimated clinical outcomes were relatively stable, reflecting the observed data from the BALLETT study (95% CI for percentage of patients with matched treatments, 12.5%-17.4%) (Figure 3). In none of the 1000 simulations was the WTP threshold of €5000 (US $5750) per matched treatment met.

### Exploratory Long-Term Threshold Analysis

**Figure 4.**
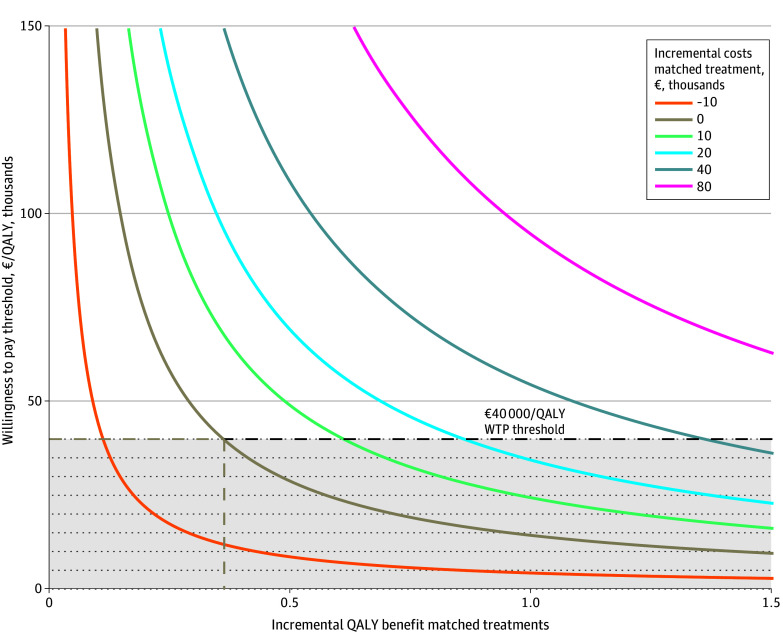
Cost-Effectiveness of Comprehensive Genomic Profiling (CGP) for Varying Incremental Costs and Benefits of CGP-Matched Treatments The colored lines present the incremental cost-effectiveness ratio of CGP compared with no diagnostics at varying incremental costs (colored lines) and varying quality-adjusted life-years (QALY) benefits of CGP-matched treatments. The conditions where CGP is cost-effective are indicated by the intersect of the plotted lines with the dotted willingness-to-pay (WTP) threshold line of €40 000 per QALY. At zero incremental CGP-matched treatment costs, the required benefit of CGP-matched treatments is shown to be 0.36 QALYs.

Incremental costs and benefits of CGP-matched treatments strongly affect CGPs’ potential cost-effectiveness. Figure 4 shows that to be cost-effective a QALY benefit of 0.36 was required from CGP-matched treatments relative to the treatment that would be provided without last-resort CGP, assuming that CGP-matched treatments did not lead to additional costs. This required QALY benefit increased to 1.36 when CGP-matched treatments would lead to additional costs of €40 000 (US $46 000) per matched treatment.

### Upfront Diagnostics Scenario

Compared with upfront SOC diagnostics, upfront CGP resulted in more patients with actionable targets (658 [81%] vs 301 [37%]) and matched treatments (308 [38%] vs 207 [26%]; ICCR, €13 936 [US $16 026]) at increased mean diagnostic costs (€2152 vs €429 [US $2475 vs US $493]) (Table). Upfront CGP costs were slightly higher compared with base-case results, mostly due to inclusion of the TSO500 homologous recombination deficiency extension for patients with a homologous recombination deficiency test indication. In the scenario only including patients with an SOC diagnostics indication, simulated diagnostic outcomes were slightly higher in both the CGP and SOC strategies. In addition, the mean diagnostic cost of the SOC strategy nearly doubled to €828 (US $952). The ICCR to match an additional treatment decreased to €10 483 (US $12 055). The 1-way sensitivity analysis yielded similar results to the base case (eFigure 4 in Supplement 1).

## Discussion

To address the scarcity of health economic evidence on CGP use, an economic evaluation was conducted alongside the observational, single-arm BALLETT study of patients with advanced cancer. The cost to identify 1 additional patient with an actionable target (€2816) was considerably lower compared with the cost to match a patient with a treatment (€14 249). Diagnostic costs and uptake of MTB recommendations were most influential in lowering these ratios. These ratios varied greatly for the 4 separately included tumor types, suggesting the importance of conducting both pancancer and tumor-specific approaches in the evaluation of CGP and clinical deployment of CGP. The upfront CGP scenario analysis showed that CGP identified more patients with actionable targets and matched treatments at higher costs compared with SOC diagnostics, resulting in a similar incremental cost per additionally matched treatment of €13 936 to the base case. Only considering patients with an indication for SOC diagnostics, mean diagnostic costs increased and more matched treatments were identified in both arms. This resulted in decreased incremental cost per additionally matched treatment with CGP (€10 483), emphasizing the importance of conducting CGP in the right population.

Previous economic analyses^14,25,26,27^ of CGP showed varying QALY and cost increases in differing populations. Our findings are difficult to compare because they use different sources and methods. We used an observational cohort that included actual on-label and investigational treatment decisions, complementing these prior model-based evaluations^14,25,26,27^ by providing insights into the diagnostic pathway that more closely reflects actual use of CGP. Diagnostic cost ratios were seldom reported in the literature. However, one study^28^ reported a diagnostic cost to identify 1 patient eligible for CGP-matched treatments ranging from €2952 to €7099, comparable to our findings.

The exploratory long-term threshold analysis highlights the importance of costs and outcomes of CGP-matched treatment. For CGP to be cost-effective, QALY benefits of CGP-matched treatments of 0.36 and 1.36 are required at zero and €40 000 incremental treatment costs, respectively. Because most CGP-matched treatments are within research, QALY benefits are uncertain. Although this approach may be beneficial, long-term evidence for CGP is scarce and mostly includes indirect comparisons, introducing biases.^3,6,29,30,31^ Additionally, a meta-analysis^32^ of randomized clinical trials in advanced cancer found a reduced risk of death with matched treatments vs SOC (hazard ratio, 0.85). Given the uncertainty surrounding CGP-matched treatment effects, high additional treatment costs may need to be avoided for last-resort CGP to be cost-effective.

CGP-matched treatments were mostly investigational, incurring limited treatment costs during trials. For example, Drug Rediscovery Protocol trial treatments, examining off-label treatments, are initially sponsored by marketing authorization holders and subsequently reimbursed via a pay-for-performance model when adequate treatment response is shown.^33^ For clinical trial enrollments, potential cost-savings are reported when prioritized over (ineffective) SOC treatments.^34,35,36,37,38^ CGP’s cost-effectiveness will change over time with new developments. Improvements can be expected if CGP is scaled up reducing sequencing costs and novel investigational treatments become available for CGP targets, improving the uptake of CGP findings. Treatment costs may, however, increase over time, as investigational treatments are approved and subsequently reimbursed at high costs, underscoring the need for iterative evaluation of CGP’s impact.

Benefits of CGP exceed treatment identification alone. For example, BALLETT study authors reported potential germline alterations in 15% of patients, potentially facilitating identification of families at risk.^8^ However, the additional costs and benefits of these findings are uncertain. Besides individual patient benefit, CGP may benefit health care systems by accelerating clinical trial accrual and, if generated data become available for secondary use, enhancing basic research and target discovery.^3^ Such benefits and related opportunity costs are complicated to incorporate in technology assessments because future value is difficult to quantify. Nevertheless, clinical evidence generation and subsequent economic evaluations on these factors are required to better understand CGP’s impact. Tools that integrate clinical, economic, and qualitative evidence adopting a holistic perspective could aid decision-makers in evaluating CGP^39^ and may assist in developing appropriate thresholds for cost-target ratios.

### Strengths and Limitations 

This study has several strengths. One strength of our study was access to patient-level prospective BALLETT study data, allowing us to inform the detailed diagnostic pathway with empirical data, unlike theoretically constructed pathways and treatment allocation in published analyses. Another strength is the use of retrospective treatment data to develop upfront diagnostics scenarios and provide insights into future implementation strategies. Our innovative approach focusing on investigational treatments provides more insights into benefits beyond on-label treatment selection, thus generating evidence for a holistic evaluation.

The study also has some limitations. A lack of comparative long-term data prevented us from demonstrating downstream consequences of CGP-matched treatments. Hence, we showed the impact of varying survival benefits and costs of CGP-matched treatments in our threshold analysis. Future research examining comparative survival effectiveness of CGP-matched treatments is required. We also had no data informing SOC diagnostics, necessitating several assumptions to inform SOC diagnostics regarding actual use, tumor development, and concordance. Developing external control arms using data from routine clinical practice could be one method to obtain useful data for future analyses.^40^ Furthermore, the impact of upfront CGP positioning on the uptake of MTB recommendations is uncertain and could affect allocation of matched treatments, both positive and negative. Earlier use of CGP may be advantageous as patients are in better health, whereas, conversely, earlier CGP targets a less selective population, potentially resulting in higher costs and lower uptake of MTB recommendations. Lastly, we had to simplify the patient pathway for the upfront diagnostics scenario, only considering whether patients received any matched treatments throughout their patient trajectories, without including repetitive testing.

## Conclusions

We conducted an empirical economic evaluation of the diagnostic pathway showing that CGP resulted in more actionable targets and matched, predominantly investigational, treatments with a diagnostic cost of €2816 to identify a patient with actionable targets and €14 249 to match one treatment in the Belgian setting. Awaiting further evidence on subsequent treatment consequences, this analysis can aid decision-makers at a local or national level as a first insight in whether to implement CGP. In addition to direct test costs and matched treatments, the broader effects of CGP on patients and health care systems warrant consideration by decision-makers.

## Notes

Supplement 1. **eTable 1.** Baseline characteristics **eTable 2.** Input parameters **eMethods 1.** Advishe validation checklist **eTable 3.** Cost calculation molecular tumor board costs **eMethods 2.** Detailed description upfront diagnostics scenario analysis **eFigure 1.** One-way sensitivity analyses of the base case analysis **eFigure 2.** Two-way sensitivity analysis of the base case analysis **eFigure 3.** Distributions of input parameters of the probabilistic sensitivity analysis for the base case **eFigure 4.** One-way sensitivity analyses of the scenario analysis
eReferences

Supplement 2.
Data Sharing Statement
