# Error in Discussion

**DOI:** 10.1001/jamanetworkopen.2025.57486

**Published:** 2026-01-09

In the Original Investigation titled “Economic Evaluation of Comprehensive Genomic Profiling in an Advanced Solid Cancer Population,”^1^ published December 11, 2025, there was an error in the Discussion. The incremental cost per additionally matched treatment should have been listed as €13 936. This article has been corrected.^1^

## References

[1] van Schaik LF, Maes B, Volders PJ, . Economic evaluation of comprehensive genomic profiling in an advanced solid cancer population. JAMA Netw Open. 2025;8(12):e2548538. doi:10.1001/jamanetworkopen.2025.48538 41379447 PMC12699362

