# Supplementary material for: Economic Evaluation of Comprehensive Genomic Profiling in an Advanced Solid Cancer Population
Source: JAMA Netw Open. 2025 Dec 11;8(12):e2548538. doi: 10.1001/jamanetworkopen.2025.48538 (PMC12699362; doi:10.1001/jamanetworkopen.2025.48538)
Supplement: Supplement 1. — eTable 1. Baseline characteristics eTable 2. Input parameters eMethods 1. Advishe validation checklist eTable 3. Cost calculation molecular tumor board costs eMethods 2. Detailed description upfront diagnostics scenario analysis eFigure 1. One-way sensitivity analyses of the base case analysis eFigure 2. Two-way sensitivity analysis of the base case analysis eFigure 3. Distributions of input parameters of the probabilistic sensitivity analysis for the base case eFigure 4. One-way sensitivity analyses of the scenario analysis eReferences [file jamanetwopen-e2548538-s001.pdf]

Supplemental Online Content

van Schaik LF, Maes B, Volders P-J, et al. Economic evaluation of comprehensive genomic profiling in an advanced solid cancer population. *JAMA Netw Open*. 2025;8(12):e2548538. doi:10.1001/jamanetworkopen.2025.48538

eTable 1. Baseline characteristics (1)

eTable 2. Input parameters

eMethods 1. Advishe validation checklist (3)

eTable 3. Cost calculation molecular tumor board costs

eMethods 2. Detailed description upfront diagnostics scenario analysis

eFigure 1. One-way sensitivity analyses of the base case analysis

eFigure 2. Two-way sensitivity analysis of the base case analysis

eFigure 3. Distributions of input parameters of the probabilistic sensitivity analysis for the base case

eFigure 4. One-way sensitivity analyses of the scenario analysis

eReferences

This supplemental material has been provided by the authors to give readers additional information about their work.

© 2025 van Schaik LF et al. *JAMA Network Open*.

eTable 1. Baseline characteristics (1)

Patient characteristics	Overall (n = 814)
**pat_sex**	
Female	452 (55.5%)
Male	362 (44.5%)
**Tumor types**	
Adrenocortical carcinoma	2 (0.2%)
Anal cancer - squamous	2 (0.2%)
Brain Glioma	38 (4.7%)
Breast invasive carcinoma	120 (14.7%)
Cancer of Unknown Primary	20 (2.5%)
Cervical carcinoma	18 (2.2%)
Cholangiocarcinoma	40 (4.9%)
Colon and rectum adenocarcinoma	86 (10.6%)
Esophageal carcinoma	17 (2.1%)
GIST	7 (0.9%)
Head and Neck squamous cell carcinoma	25 (3.1%)
Kidney cancer	20 (2.5%)
Liver hepatocellular carcinoma	4 (0.5%)
Lung cancer	76 (9.3%)
Mesothelioma	10 (1.2%)
Miscellaneous cancer	11 (1.4%)
Neuroendocrine tumor, non-lung, non-pancreas	16 (2.0%)
Ovarian carcinoma	36 (4.4%)
Pancreas cancer	38 (4.7%)
Penile cancer	2 (0.2%)
Prostate adenocarcinoma	25 (3.1%)
Salivary gland carcinoma	7 (0.9%)
Sarcoma	53 (6.5%)
Skin cancer	36 (4.4%)
Stomach adenocarcinoma	17 (2.1%)
Testicular Cancer	2 (0.2%)
Thymoma	6 (0.7%)
Thyroid carcinoma	18 (2.2%)
Urothelial Carcinoma	40 (4.9%)
Uterus cancer	11 (1.4%)
Uveal Melanoma	7 (0.9%)
Vulva Carcinoma	4 (0.5%)
**age_inclusion**	
Mean (SD)	60.8 (12.3)
Median [Min, Max]	62.0 [21.0, 88.0]

© 2025 van Schaik LF et al. *JAMA Network Open*.

Patient characteristics	Overall (n = 814)
**Meta_YesNo**	
No (locally advanced)	101 (12.4%)
Yes	713 (87.6%)
**factor(ECOG_atIncl)**	
0	149 (18.3%)
1	373 (45.8%)
2	38 (4.7%)
Not available	254 (31.2%)

eTable 2. Input parameters

Input parameters for the base case

Input parameters	CGP	distribution for PSA	Estimation method
probability of successful diagnostics	0,93	Beta	Number of patients by which CGP was successful, following definitions of the clinical BALLETT study / total number of patients where CGP was conducted
probability of repeat diagnostics	0,05	Beta	Number of patients with repeat CGP conducted / total number of patients
Probability of actionable target	0,82	Beta	Number of patients with actionable targets / number of patients with successful CGP
Probability of MTB recommendation	0,85	Beta	Number of patients with MTB recommendations / number of patients with actionable targets
Probability of treatment distribution for patients with MTB recommendations			Number of patients with type x treatment / number of patients with MTB recommendations
On-label matched treatment	0,05	Dirichlet	
investigational or off-label matched treatment	0,18	Dirichlet	
Other treatment	0,48	Dirichlet	
No treatment	0,29	Dirichlet	
Probability of treatment distribution for patients without MTB recommendations			Number of patients with type x treatment / number of patients without MTB recommendations
On-label matched treatment	0	Dirichlet	
investigational or off-label matched treatment	0	Dirichlet	
Other treatment	0,43	Dirichlet	
No treatment	0,57	Dirichlet	

© 2025 van Schaik LF et al. *JAMA Network Open*.

Input parameters	CGP	distribution for PSA	Estimation method
Probability of treatment distribution for patients without actionable targets			Number of patients with type x treatment / number of patients without actionable targets
On-label matched treatment	0	Dirichlet	
investigational or off-label matched treatment	0	Dirichlet	
Other treatment	0,39	Dirichlet	
No treatment	0,61	Dirichlet	
Probability of treatment distribution for patients with unsuccessful CGP			Number of patients with type x treatment / number of patients with unsuccessful CGP
On-label matched treatment	0	Dirichlet	
investigational or off-label matched treatment	0	Dirichlet	
Other treatment	0,08	Dirichlet	
No treatment	0,92	Dirichlet	
Cost of CGP	€1831,94	Gamma	Informed by microcosting conducted as part of the BALLETT study (2)
Cost of MTB	€209,50	Gamma	See Supplement eTable 4 for calculation

© 2025 van Schaik LF et al. *JAMA Network Open*.

eMethods 1. Advishe validation checklist (3)

Assessment of the Validation Status of Health-Economic decision models (AdViSHE) is a questionnaire that modelers can complete to report on the efforts performed to improve the validation status of their health-economic (HE) decision model. It is not intended to replace validation by model users but rather to inform the direction of validation efforts and to provide a baseline for replication of the results. In addition to using it after a model is finished, the modelers can use AdViSHE to guide validation efforts during the modelling process.
The modelers are asked to comment on the validation efforts performed while building the underlying HE decision model and afterward. Many of the questions simply refer to the model documentation. The AdViSHE is divided into 5 parts, each covering an aspect of validation:

- Part A: Validation of the conceptual model (2 questions)
- Part B: Input data validation (2 questions)
- Part C: Validation of the computerized model (4 questions)
- Part D: Operational validation (4 questions)
- Part E: Other validation techniques (1 question)

No final validation score is calculated because the assessment of the answers and the overall validation effort are left to the model users. It is assumed that the model has been built according to prevailing modelling and reporting guidelines. For instance, the model builders would presumably adhere to the ISPOR-SMDM (International Society for Pharmacoeconomics and Outcomes Research—Society for Medical Decision-Making) Modeling Good Research Practices (Caro et al, 2012)[E1] and/or CHEERS (Consolidated Health Economic Evaluation Reporting Standards) Statement (Husereau et al, 2013).[E2] Some questions may not be applicable to a particular model. If this is the case, the model builder should take the opt-out option and provide a justification of why this item is not deemed applicable.

Part A: Validation of the conceptual model (2 questions)

Part A discusses techniques for validating the conceptual model. A conceptual model describes the underlying system (eg, progression of disease) using a mathematical, logical, verbal, or graphical representation. Please indicate where the conceptual model and its underlying assumptions are described and justified.

The conceptual model is described and justified in the methods section under 2.2 model description. The assumptions made for the scenario analysis are described in 2.4.2 Upfront diagnostic scenario analysis, and in eMethods 3, supplementary 1.
**A1/ Face validity testing (conceptual model):** Have experts been asked to judge the appropriateness of the conceptual model?<br>If yes, please provide information on the following aspects:<br>-<br>Who are these experts?<br>-<br>What is your justification for considering them experts?<br>-<br>To what extent do they agree that the conceptual model is appropriate?<br>If no, please indicate why not.
**Yes, namely:**<br>**-MD Brigitte Maes, PhD, clinical biologist**<br><br>**Justification for this expert: Clinical biologists have knowledge about molecular diagnostics and the clinical consequences of genomic profiling. This expert in particular was involved in the BALLETT study and had extensive knowledge on the clinical study data, and the patients included in the BALLETT study. Furthermore, this expert had knowledge on the molecular diagnostics indicated as standard of care for patients.**<br>**The constructed decision tree was presented in a meeting with this expert and small changes were made in response to the proposed feedback (mainly, the inclusion of MTB recommendations in the decision tree).**

© 2025 van Schaik LF et al. *JAMA Network Open*.

Aspects to judge include appropriateness to represent the underlying clinical process/disease (eg, disease stages, physiological processes); and appropriateness for economic evaluation (eg, comparators, perspective, costs covered).

A2/ Cross validity testing (conceptual model): Has this model been compared with other conceptual models found in the literature or clinical textbooks?
If yes, please indicate where this comparison is reported.
If no, please indicate why not.

No, the model was not compared with existing models. This is due to the novel tumor agnostic approach that was taken for this model. In addition, the model focused on the diagnostic pathway and did not model in detail disease processes. As a consequence, we judged that face validation with the clinical data (does the model present the diagnostic process as is observed in the data) was sufficient for this model. Additionally, we did not identify similar models.

Part B: Input data validation (2 questions)

Part B discusses techniques to validate the data serving as input in the model. These techniques are applicable to all types of models commonly used in HE modelling.
Please indicate where the description and justification of the following aspects are given:

- Search strategy
- Data sources, including descriptive statistics
- Reasons for inclusion of these data sources
- Reasons for exclusion of other available data sources
- Assumptions that have been made to assign values to parameters for which no data were available;
- Distributions and parameters to represent uncertainty
- Data adjustments: mathematical transformations (eg, logarithms, squares); treatment of outliers; treatment of missing data; data synthesis (indirect treatment comparison, network meta-analysis); calibration; and the like

- **The data source (BALLETT study) is described in the methods in section 2.1 and in a separate publication (listed in the methods). In addition, a table 1 of the study population is presented in the Supplement.**
- **Costs were included from a micro-costing study (published in the European Can.Heal consortium). This microcosting was utilized as it followed a published cost framework (Pasmans, 2019) and the microcosting was conducted in the Belgian context with the correct tests.**
- **Utilities were not included, as we only included the diagnostic time horizon.**
- **Assumptions that were made for input parameters for which we had no data available are described in the Supplement Input parameter table.**
- **Distributions were estimated to capture the uncertainty of the parameters. For probabilities, beta distributions were estimated, except for the probability to identify treatment options, for which dirichlet distributions were estimated as more than two treatment types could be identified. For costs, gamma distributions were estimated. If no uncertainty was available, a SE of 20% was assumed to estimate the distribution.**

B1/ Face validity testing (input data): Have experts been asked to judge the appropriateness of the input data?
If yes, please provide information on the following aspects:
-Who are these experts?
-What is your justification for considering them experts?
-To what extent do they agree that appropriate data has been used?
If no, please indicate why not.

Yes, namely:
- MD Brigitte Maes, PhD, clinical biologist
Justification for this expert: Clinical biologists have knowledge about molecular diagnostics and the clinical consequences of genomic profiling. This expert in particular was involved in the BALLETT study and had extensive knowledge on the clinical study data, and the patients included in the BALLETT study. Furthermore, this expert had knowledge on the molecular diagnostics indicated as standard of care for patients.

© 2025 van Schaik LF et al. *JAMA Network Open*.

Input data was discussed in a meeting. In this meeting, alignment between the clinical data analysis and health economic analysis was discussed on input parameters. In the future implementation scenario, the use of retrospective patient level treatment data was discussed. Agreement was found on the use of input parameters from the data, and the estimation of diagnostic cost data.

Aspects to judge may include but are not limited to potential for bias; generalizability to the target population; availability of alternative data sources; any adjustments made to the data.

B2/ Model fit testing: When input parameters are based on regression models, have statistical tests been performed?
If yes, please indicate where the description, the justification and the outcomes of these tests are reported.
If no, please indicate why not.

Not applicable

Examples of regression models include but are not limited to disease progression based on survival curves; risk profiles using regression analysis on a cohort; local cost estimates based on multilevel models; meta-regression; quality-of-life weights estimated using discrete choice analysis; mapping of disease-specific quality-of-life weights to utility values. Examples of tests include but are not limited to comparing model fit parameters (R^2, Akaike information criterion [AIC], Bayesian information criterion [BIC]); comparing alternative model specifications (covariates, distributional assumptions); comparing alternative distributions for survival curves (Weibull, lognormal, logit); testing the numerical stability of the outcomes (sufficient number of iterations); testing the convergence of the regression model; visually testing model fit and/or regression residuals.

Part C: Validation of the computerized model (4 questions)

Part C discusses various techniques for validating the model as it is implemented in a software program. If there are any differences between the conceptual model (part A) and the final computerized model, please indicate where these differences are reported and justified.

Not applicable

C1/ External review: Has the computerized model been examined by modelling experts?
If yes, please provide information on the following aspects:
-Who are these experts?
-What is your justification for considering them experts?
-Can these experts be qualified as independent?
-Please indicate where the results of this review are reported, including a discussion of any unresolved issues.
If no, please indicate why not.

The model was checked by E. Krijkamp, followed with a discussion. She was considered an expert as she developed the DARTH R framework which was followed to develop the model. She has extensive knowledge of different model types and the R methodology that was used. The discussion identified several small terminologies that might be adapted, however no issues on modeling code were identified.

Aspects to judge may include but are not limited to absence of apparent bugs; logical code structure optimized for speed and accuracy; appropriate translation of the conceptual model.

C2/ Extreme value testing: Has the model been run for specific, extreme sets of parameter values in order to detect any coding errors?
If yes, please indicate where these tests and their outcomes are reported.
If no, please indicate why not.

Yes, one and two way sensitivity analyses have been conducted varying values to extremes, and model outcomes changed accordingly. No deviating outcomes were observed.

Examples include but are not limited to 0 and extremely high (background) mortality; extremely beneficial, extremely detrimental, or no treatment effect; 0 or extremely high treatment or health care costs.

© 2025 van Schaik LF et al. *JAMA Network Open*.

C3/ Testing of traces: Have patients been tracked through the model to determine whether its logic is correct?
If yes, please indicate where these tests and their outcomes are reported.
If no, please indicate why not.

Yes, traces of patients were tested by tracking the weights in the decision tree. The decision tree weights are saved in an R markdown document by the authors and can be obtained upon request.

In cohort models, this would involve listing the number of patients in each disease stage at one, several, or all time points (eg, Markov traces). In individual patient simulation models, this would involve following several patients throughout their natural disease progression.

C4/ Unit testing: Have individual submodules of the computerized model been tested?
If yes, please provide information on the following aspects:
-Was a protocol that describes the tests, criteria, and acceptance norms defined beforehand?
-Please indicate where these tests and their outcomes are reported.
If no, please indicate why not.

The model did not consists of individual submodules, as the model involves one decision tree.

Examples include but are not limited to turning submodules of the program on and off; altering global parameters; testing messages (eg, warning against illegal or illogical inputs), drop-down menus, named areas, switches, labelling, formulas and macros; removing redundant elements.

Part D: Operational validation (4 questions)

Part D discusses techniques used to validate the model outcomes.

D1/ Face validity testing (model outcomes): Have experts been asked to judge the appropriateness of the model outcomes?
If yes, please provide information on the following aspects:
-Who are these experts?
-What is your justification for considering them experts?
-To what extent did they conclude that the model outcomes are reasonable?
If no, please indicate why not.

Yes, the model appropriateness was judged by the experts described in A1 and C1. Appropriateness of model outcomes was extensively discussed with these experts during the development phase of the model.
In addition, the outcomes of the model were compared with clinical outcomes, which were similar.

Outcomes may include but are not limited to:(quality-adjusted) life years; deaths; hospitalizations; total costs.

D2/ Cross validation testing (model outcomes): Have the model outcomes been compared with the outcomes of other models that address similar problems?
If yes, please provide information on the following aspects:
-Are these comparisons based on published outcomes only, or did you have access to the alternative model?
-Can the differences in outcomes between your model and other models be explained?
-Please indicate where this comparison is reported, including a discussion of the comparability with your model.
If no, please indicate why not.

No, because no models are available that address the same context in a similar method as we do. The choice to include a tumor agnostic population, use empirical data driven last resort diagnostics and focus on diagnostic outcomes compared to survival made this study non comparable to other published analyses, which were model based and generally excluded the last resort effects (investigational treatments).
Outcomes were compared and discussed in the discussion section by a retrospective observational study examining costs and outcomes of CGP, and results were similar. This study was however not an economic model.

© 2025 van Schaik LF et al. *JAMA Network Open*.

Other models may include models that describe the same disease, the same intervention, and/or the same population.

D3/ Validation against outcomes using alternative input data: Have the model outcomes been compared with the outcomes obtained when using alternative input data?
If yes, please indicate where these tests and their outcomes are reported.
If no, please indicate why not.

This has not been conducted, as the results are highly specific to the population and health-care specific context. Therefore, it is of little use to perform these comparisons. In addition, our model does not extrapolate, and can therefore be compared to the clinical outcomes of the empirical study that was conducted.

Alternative input data can be obtained by using different literature sources or datasets, but can also be constructed by splitting the original dataset in 2 parts, and using one part to calculate the model outcomes and the other part to validate against.

D4/ Validation against empirical data: Have the model outcomes been compared with empirical data?
If yes, please provide information on the following aspects:
-Are these comparisons based on summary statistics, or patient-level datasets?
-Have you been able to explain any difference between the model outcomes and empirical data?
-Please indicate where this comparison is reported.
If no, please indicate why not.
D4.A/ Comparison against the data sources on which the model is based (dependent validation).

A face validation check was conducted to examine whether similar outcomes were identified. This was not confirmed with a statistical test.

D4.B/ Comparison against a data source that was not used to build the model (independent validation).

This was not conducted.

Part E: Other validation techniques (1 question)

E1/ Other validation techniques: Have any other validation techniques been performed?
If yes, indicate where the application and outcomes are reported, or else provide a short summary here.

We have organized a walk-through with the clinician described under section A. In addition, the model code was checked by a member of our research group with a health economic background.

Examples of other validation techniques: structured walk-throughs (guiding others through the conceptual model or computerized program step-by-step); naive benchmarking (back-of-the-envelope calculations); heterogeneity tests; double programming (2 model developers program components independently and/or the model is programmed in 2 different software packages to determine whether the same results are obtained).

© 2025 van Schaik LF et al. *JAMA Network Open*.

eTable 3. Cost calculation molecular tumor board costs

**Number of patients / MTB**	**10**			
**Duration MTB (h)**	**1**			
**Salary costs of specialists**	**€/hour**	**€/MTB**	**n / MTB**	**€/patient**
Molecular biologist	60,00 €	60,00 €	5	€ 30,00
Bio-information specialist	65,00 €	65,00 €	3	€ 19,50
medical specialist	200,00 €	200,00 €	8	€ 160,00
**Total**				**€ 209,50**

Following expert input on molecular tumor board practices and salaries in the Belgian setting.

MTB Molecular tumor board. (h) hour.

© 2025 van Schaik LF et al. *JAMA Network Open*.

eMethods 2. Detailed description upfront diagnostics scenario analysis

This eMethods contains further details and assumptions relevant for the upfront diagnostics scenario analysis. This analysis compares upfront comprehensive genomic profiling (CGP) with upfront standard of care (SOC) diagnostics. This analysis is based on the clinical data obtained from the BALLETT study, and retrospective patient-level data that was obtained for patients included in the BALLETT study. This retrospective data included the types of treatments patients had received during SOC, before BALLETT enrollment. Included outcomes are diagnostic costs, patients with actionable targets, and patients with matched treatments. In the table below, all clinical input parameters and methods of calculation are described for both the SOC and CGP arm.

The assumptions that were made for this analysis are listed below:

- We assumed that molecular findings of a patients' tumor remained stable over time. This is documented by van de Haar and colleagues, who examined evolving tumor landscapes over time and noted few noticeable differences. This assumption was required as we only had CGP findings in the last resort setting. This assumption allowed us to use these findings to inform upfront CGP (4).
- We assumed comparable test performance between SOC diagnostics and CGP. This was based on a validation study of the TSO500 by Froyen, et al. showing that the TSO500 had >99% accuracy and precision for all variant types. This assumption was required as we did not have SOC sequencing results. By assuming similar performance, we could filter the CGP findings with targets that are included in SOC guidelines. With SOC testing, only these targets would have been identified (5).
- We assumed that SOC diagnostics would be employed following SOC diagnostic Compermed guidelines. As we had no data for actual SOC utilization, we had to assume that SOC NGS would have been deployed following existing guidelines.
- We assumed that moving CGP to the upfront setting had a net zero effect on the uptake of molecular tumor board (MTB) recommendations. The uptake of MTB recommendations could be influenced both positively and negatively with this setting change. A higher uptake could be observed as a result of a healthier population, as in the BALLETT study many patients did not follow MTB recommendations due to fast deterioration of their health status. On the other hand, upfront CGP would be provided to more patients compared to the selective BALLETT cohort, and many of those patients may in the end not have a need for CGP-matched treatments, which could negatively affect the uptake of MTB recommendations. In this analysis, we assumed a net zero effect as we did not identify reliable sources that indicate the uptake of upfront CGP in the Belgian setting.

eMethods 2 Table 1. Clinical input parameters for upfront diagnostics scenario analysis

A: CGP input parameters scenario analysis

Input parameters	CGP	distribution for PSA	Assumption
probability of successful diagnostics	0,95	Beta	Informed by clinical opinion from laboratory technician, based on clinical experiences within the validation studies for the TSO500 (5).
probability of repeat diagnostics	0,05	Beta	Informed by clinical opinion from laboratory technician.
Probability of actionable target	0,85	Beta	Number of patients with actionable targets / number of patients with successful CGP. All patients that received targeted treatments before BALLETT enrollment were automatically classified as actionable.
Probability of MTB recommendation	0,82	Beta	Number of patients with MTB recommendations / number of patients with actionable targets.
Probability of treatment			Informed by both treatments administered after and before BALLETT enrollment. Number of patients with type x treatment / number of patients

© 2025 van Schaik LF et al. *JAMA Network Open*.

Input parameters	CGP	distribution for PSA	Assumption
distribution for patients with MTB recommendations			with MTB recommendations. If a patient received both on-label and investigational matched treatment, the patient was classified in the on-label & investigational matched treatment group. Not all patients had complete follow-up in the BALLETT cohort, and stratified mean imputation was used to account for this.
On-label matched treatment	0,28	Dirichlet	
investigational or off-label matched treatment	0,13	Dirichlet	
On-label matched treatment & investigational or off-label matched treatment	0,03	Dirichlet	
Other treatment	0,56	Dirichlet	
Probability of treatment distribution for patients without MTB recommendations			Informed by both treatments administered after and before BALLETT enrollment. Number of patients with type x treatment / number of patients without MTB recommendations. In this case, while not having an MTB recommendation from CGP, patients still could have on-label targeted treatments administered before BALLETT enrollment.
On-label matched treatment	0,43	Dirichlet	
investigational or off-label matched treatment	0	Dirichlet	
On-label matched treatment & investigational or off-label matched treatment	0	Dirichlet	
Other treatment	0,57	Dirichlet	
Probability of treatment distribution for patients without actionable targets			Informed by both treatments administered after and before BALLETT enrollment. Number of patients with type x treatment / number of patients without actionable targets.
On-label matched treatment	0	Dirichlet	
investigational or off-label matched treatment	0	Dirichlet	
On-label matched treatment & investigational or off-label matched treatment	0	Dirichlet	
Other treatment	1	Dirichlet	

© 2025 van Schaik LF et al. *JAMA Network Open*.

Input parameters	CGP	distribution for PSA	Assumption
Probability of treatment distribution for patients with unsucessful CGP			Assumption that no on-label treatment can be matched when you have no successful sequencing
On-label matched treatment	0	Dirichlet	
investigational or off-label matched treatment	0	Dirichlet	
On-label matched treatment & *investigational or off-label matched treatment*	0	Dirichlet	
Other treatment	1	Dirichlet	
probability of use addon for HRD	0,03	beta	Percentage of patients with high grade serous ovarian cancer in BALLETT cohort

© 2025 van Schaik LF et al. *JAMA Network Open*.

B: SOC input parameters scenario analysis

Input parameters	SOC	distribution for PSA	Assumption
probability of successful diagnostics	0,98	Beta	Informed by clinical opinion from laboratory technician, based on clinical experiences with targeted NGS panels.
probability of repeat diagnostics	0,05	Beta	Informed by clinical opinion from laboratory technician and similar repeat diagnostics probability assumed as CGP
Probability of actionable target	0,38	Beta	Informed by BALLETT patients with actionable targets, filtering their CGP results with targets present in SOC guidelines. All patients that received targeted treatments before BALLETT enrollment were automatically classified as actionable.
Probability of MTB recommendation	NA		
Probability of treatment distribution for patients with actionable targets			
On-label matched treatment	0,69	Dirichlet	Number of BALLETT patients with actionable targets with type x treatment (only including treatments provided before BALLETT enrollment) / all patients with actionable targets.<br>It was assumed that no matched investigational treatments were provided before BALLETT enrollment.
investigational or off-label matched treatment	0	Dirichlet	
On-label matched treatment <u>*&*</u> *investigational or off-label matched treatment*	0	Dirichlet	
Other treatment	0,31	Dirichlet	
Probability of treatment distribution for patients without actionable targets			Number of BALLETT patients without actionable targets with type x treatment (only including treatments provided before BALLETT enrollment) / all patients without actionable targets.<br>It was assumed that no matched investigational treatments were provided before BALLETT enrollment.
On-label matched treatment	0	Dirichlet	
investigational or off-label matched treatment	0	Dirichlet	

© 2025 van Schaik LF et al. *JAMA Network Open*.

Input parameters	SOC	distribution for PSA	Assumption
probability of successful diagnostics	0,98	Beta	Informed by clinical opinion from laboratory technician, based on clinical experiences with targeted NGS panels.
probability of repeat diagnostics	0,05	Beta	Informed by clinical opinion from laboratory technician and similar repeat diagnostics probability assumed as CGP
Probability of actionable target	0,38	Beta	Informed by BALLETT patients with actionable targets, filtering their CGP results with targets present in SOC guidelines. All patients that received targeted treatments before BALLETT enrollment were automatically classified as actionable.
Probability of MTB recommendation	NA		
Probability of treatment distribution for patients with actionable targets			
On-label matched treatment	0,69	Dirichlet	Number of BALLETT patients with actionable targets with type x treatment (only including treatments provided before BALLETT enrollment) / all patients with actionable targets.<br>It was assumed that no matched investigational treatments were provided before BALLETT enrollment.
investigational or off-label matched treatment	0	Dirichlet	
On-label matched treatment & investigational or off-label matched treatment	0	Dirichlet	
Other treatment	0,31	Dirichlet	
On-label matched treatment & investigational or off-label matched treatment	0	Dirichlet	
Other treatment	1	Dirichlet	
Probability of treatment distribution for			Assumption that no on-label treatment can be matched when you have no successful sequencing

© 2025 van Schaik LF et al. *JAMA Network Open*.

Input parameters	SOC	distribution for PSA	Assumption
probability of successful diagnostics	0,98	Beta	Informed by clinical opinion from laboratory technician, based on clinical experiences with targeted NGS panels.
probability of repeat diagnostics	0,05	Beta	Informed by clinical opinion from laboratory technician and similar repeat diagnostics probability assumed as CGP
Probability of actionable target	0,38	Beta	Informed by BALLETT patients with actionable targets, filtering their CGP results with targets present in SOC guidelines. All patients that received targeted treatments before BALLETT enrollment were automatically classified as actionable.
Probability of MTB recommendation	NA		
Probability of treatment distribution for patients with actionable targets			
On-label matched treatment	0,69	Dirichlet	Number of BALLETT patients with actionable targets with type x treatment (only including treatments provided before BALLETT enrollment) / all patients with actionable targets.<br>It was assumed that no matched investigational treatments were provided before BALLETT enrollment.
investigational or off-label matched treatment	0	Dirichlet	
On-label matched treatment & investigational or off-label matched treatment	0	Dirichlet	
Other treatment	0,31	Dirichlet	
patients with unsucessful CGP			
On-label matched treatment	0	Dirichlet	
investigational or off-label matched treatment	0	Dirichlet	
On-label matched treatment & investigational or off-label matched treatment	0	Dirichlet	

© 2025 van Schaik LF et al. *JAMA Network Open*.

Input parameters	SOC	distribution for PSA	Assumption
probability of successful diagnostics	0,98	Beta	Informed by clinical opinion from laboratory technician, based on clinical experiences with targeted NGS panels.
probability of repeat diagnostics	0,05	Beta	Informed by clinical opinion from laboratory technician and similar repeat diagnostics probability assumed as CGP
Probability of actionable target	0,38	Beta	Informed by BALLETT patients with actionable targets, filtering their CGP results with targets present in SOC guidelines. All patients that received targeted treatments before BALLETT enrollment were automatically classified as actionable.
Probability of MTB recommendation	NA		
Probability of treatment distribution for patients with actionable targets			
On-label matched treatment	0,69	Dirichlet	Number of BALLETT patients with actionable targets with type x treatment (only including treatments provided before BALLETT enrollment) / all patients with actionable targets.<br>It was assumed that no matched investigational treatments were provided before BALLETT enrollment.
investigational or off-label matched treatment	0	Dirichlet	
On-label matched treatment <u>*&*</u> *investigational or off-label matched treatment*	0	Dirichlet	
Other treatment	0,31	Dirichlet	
Other treatment	1	Dirichlet	

© 2025 van Schaik LF et al. *JAMA Network Open*.

Test cost estimation

For SOC diagnostics, a weighted average diagnostic cost is estimated by multiplying the resource usage of NGS, weighted by the distribution of cancer subtypes in the BALLETT cohort, with test cost estimations.

Resource use of NGS was determined for each tumor type, and subtype if relevant, in the BALLETT study population. The guidelines from ComPerMed (https://www.compermed.be/en/workflows), which include recommendations for molecular testing in various cancer subtypes, as well as reimbursement schemes for molecular testing (https://www.riziv.fgov.be/nl/professionals/verzorgingsinstellingen-en-diensten/laboratoria/hemato-oncologie-terugbetaling-van-moleculair-biologische-testen-met-next-generation-sequencing) , were reviewed to inform the utilization of NGS resources. If molecular profiling was only indicated to confirm diagnostically challenging cases, no NGS utilization was assumed. The estimated NGS resource utilization was confirmed with a clinical pathologist from a Belgian hospital. In tumor types were guidelines were absent, this clinical pathologist provided input to estimate NGS resource utilization in clinical practice. The cost estimation includes upfront testing and excludes repetitive testing after treatment progression.

The indicated tests included DNA NGS, DNA + RNA NGS, and HRD testing. Costs for the DNA, and DNA + RNA NGS were obtained from a cost estimation including 11 DNA NGS panels and 8 DNA + RNA NGS panel combinations from different laboratories in Belgium (Table 1) (https://zenodo.org/records/15047664, CAN.HEAL WP9: Deliverable Report D9.1: National Precision Initiatives). These cost estimations followed the same costing method as the cost estimation performed for CGP. The costs for HRD testing were obtained from the Compermed guideline. While not included in the micro-costing of CGP, detecting HRD with the TSO500 required the use of an add-on HRD kit, estimated at €150 by the BALLETT study investigator. This amount was multiplied with the proportion of patients who required HRD testing according to the guideline and added to the cost of CGP.

eMethods 2 Table 2. Estimated weighted mean standard of care test costs

Test	mean cost [SD](€)	Percentage of patients receiving NGS testing (%)	Weighted cost (€)
DNA NGS	€666 [€147] (2)	37%	€249
DNA + RNA NGS	€1.067 [€460] (2)	11%	€119
HRD	€1.200[a, b] [-] (6)	3%	€40
Weighted mean cost			€408

[a] Based on ComPerMed reimbursed rate for HRD testing.

[b] Only one estimate, therefore this estimate was used as average.

The weighted average cost for standard-of-care upfront NGS diagnostics was calculated by summing the products of the fraction of patients undergoing each type of test and the corresponding estimated test costs, described in the formula below:

$$Weighted\ SOC\ cost = \left(C_{DNA} * \frac{n_{tumor\ types\ DNA}}{n_{total\ cohort}}\right) + \left(C_{DNA_{RNA}} * \frac{n_{tumor\ types\ DNA_{RNA}}}{n_{total\ cohort}}\right) + \left(C_{HRD} * \frac{n_{tumor\ types\ HRD}}{n_{total\ cohort}}\right)$$

$\mathbf{C_{DNA}}$ = average DNA NGS costs, $\mathbf{n_{tumor\ types\ DNA}}$ = number of patients with DNA NGS indication, $\mathbf{n_{total\ cohort}}$ = total number of patients, $\mathbf{C_{DNA\_RNA}}$ = average DNA + RNA NGS costs, $\mathbf{n_{tumor\ types\ DNA\_RNA}}$ = number of patients with DNA + RNA NGS indication, $\mathbf{C_{HRD}}$ = average HRD test costs, $\mathbf{n_{tumor\ types\ HRD}}$ = number of patients with HRD test indication.

Probabilistic sensitivity analysis

To incorporate the uncertainty of the weighted SOC costs in the probabilistic sensitivity analysis, 10.000 weighted SOC cost estimations were calculated by sampling cost estimations from fitted gamma distributions and by sampling

© 2025 van Schaik LF et al. *JAMA Network Open*.

proportions of patients receiving DNA NGS, RNA + DNA NGS, or HRD testing from fitted beta distributions. For HRD, which only had one cost estimation, a standard error of 20% was assumed.

eMethods 2 Table 3 Baseline characteristics of SOC diagnostics indicated cohort

**Patient characteristics**	Overall (n=422)
**pat_sex**	
Female	239 (56.6%)
Male	183 (43.4%)
**Dx_type**	
Brain Glioma	37 (8.8%)
Breast invasive carcinoma	66 (15.6%)
Cancer of Unknown Primary	20 (4.7%)
Colon and rectum adenocarcinoma	86 (20.4%)
GIST	7 (1.7%)
Lung cancer	71 (16.8%)
Ovarian carcinoma	27 (6.4%)
Pancreas cancer	35 (8.3%)
Prostate adenocarcinoma	25 (5.9%)
Skin cancer	30 (7.1%)
Uterus cancer	11 (2.6%)
Uveal Melanoma	7 (1.7%)
**age_inclusion**	
Mean (SD)	61.6 (11.8)
Median [Min, Max]	63.0 [23.0, 85.0]
**Meta_YesNo**	
No (locally advanced)	51 (12.1%)
Yes	371 (87.9%)
**as.numeric(Meta_number)**	
Mean (SD)	2.37 (1.52)
Median [Min, Max]	2.00 [1.00, 20.0]
Missing	54 (12.8%)
**as.numeric(TreatmentLines_number)**	
Mean (SD)	2.12 (1.85)
Median [Min, Max]	2.00 [0, 11.0]
**factor(ECOG_atIncl)**	
0	71 (16.8%)
1	193 (45.7%)
2	18 (4.3%)
Not available	140 (33.2%)

© 2025 van Schaik LF et al. *JAMA Network Open*.

eFigure 1. One-way sensitivity analyses of the base case analysis

In this one way sensitivity analysis, input parameters are varied and plotted against the average net monetary benefit (NMB) per patient. The NMB uses a willingness to pay defined as €5,000 / matched treatment. When the NMB > 0, the graph indicates that for that specific input parameter the benefits outweigh the costs.

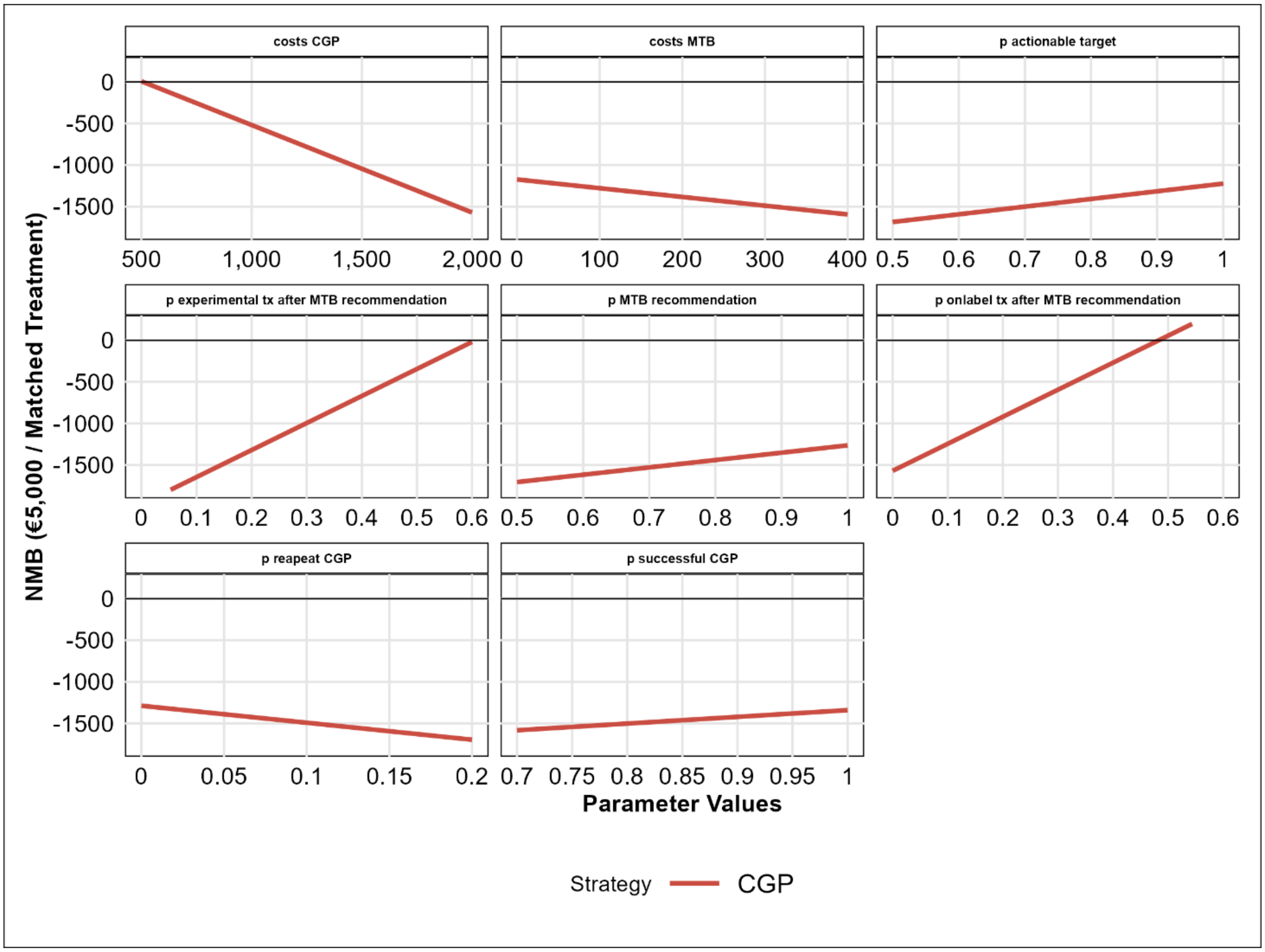

NMB, net monetary benefit, CGP comprehensive genomic profiling, MTB molecular tumor board, p probability, tx treatment p probability tx treatment

© 2025 van Schaik LF et al. *JAMA Network Open*.

eFigure 2. Two-way sensitivity analysis of the base case analysis

Two-way sensitivity analysis varying cost of CGP and the uptake of MTB recommendations to any type of matched treatment. The NMB is defined as €5,000 / matched treatments, and the green area shows at what parameter combinations a positive NMB is achieved.

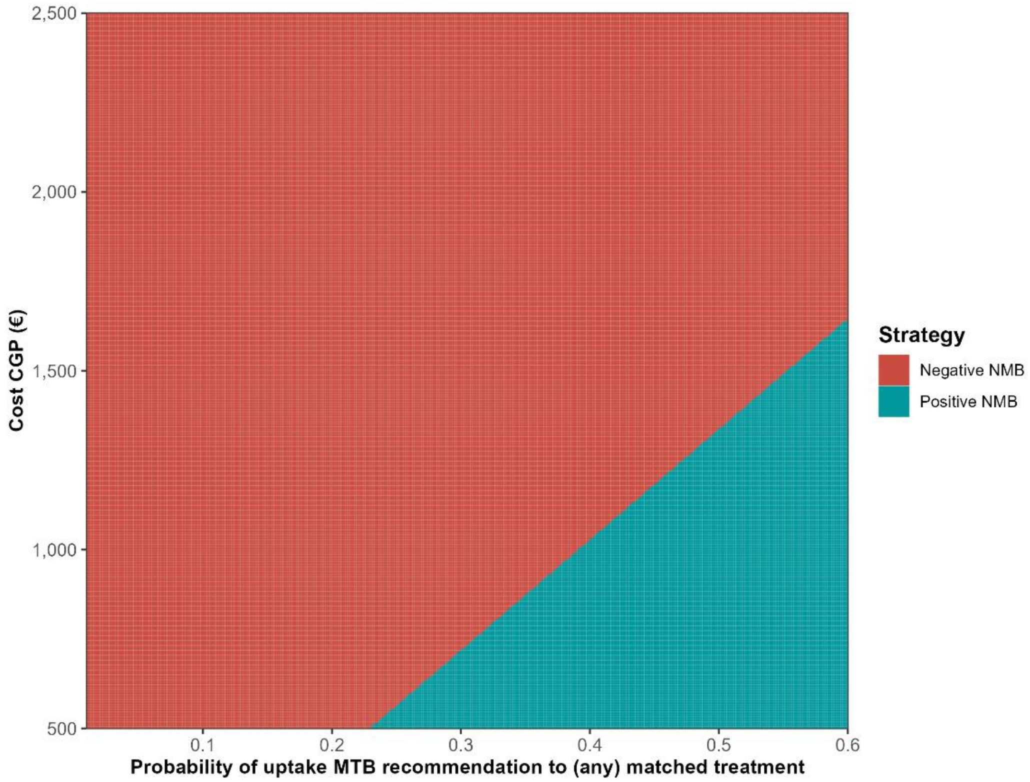

CGP Comprehensive genomic profiling, MTB molecular tumor board, NMB Net monetary benefit

© 2025 van Schaik LF et al. *JAMA Network Open*.

eFigure 3. Distributions of input parameters of the probabilistic sensitivity analysis for the base case

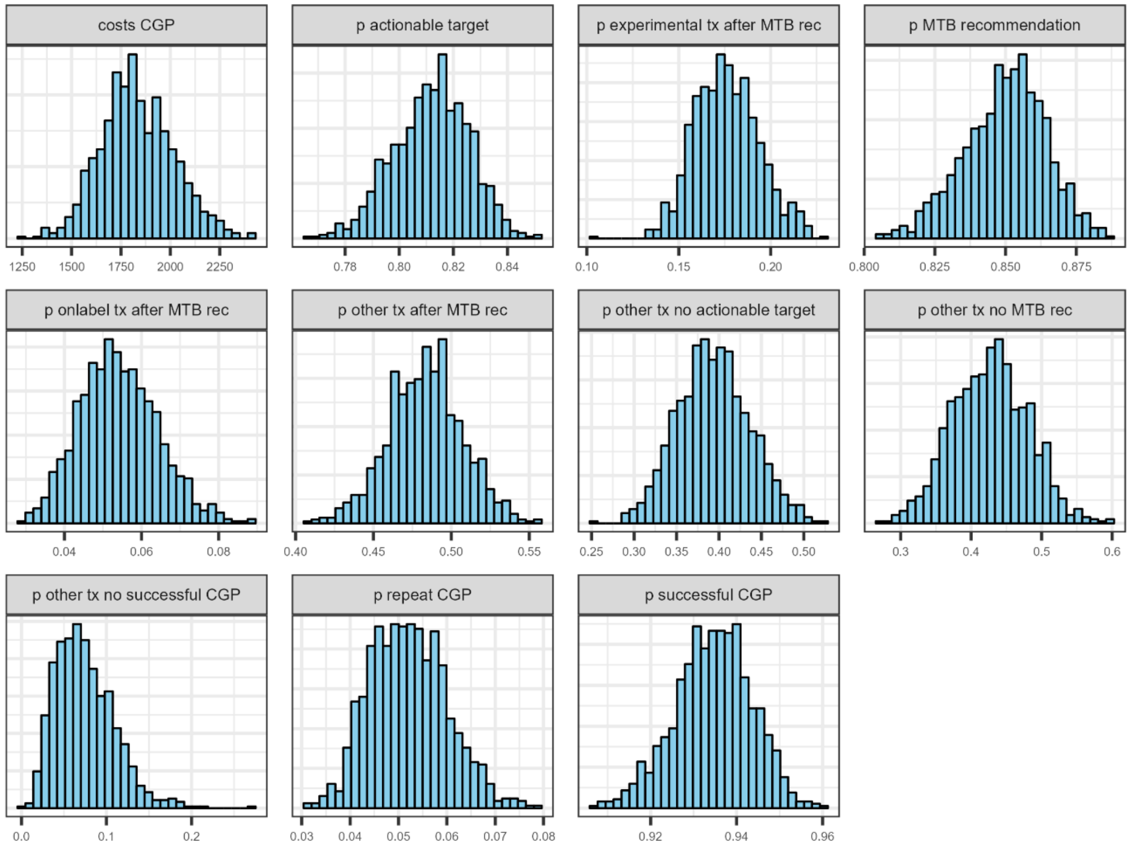

CGP comprehensive genomic profiling, p probability, tx treatment, rec recommendation, MTB molecular tumor board.

© 2025 van Schaik LF et al. *JAMA Network Open*.

eFigure 4. One-way sensitivity analyses of the scenario analysis

In this one way sensitivity analysis, input parameters are varied and plotted against the average net monetary benefit (NMB) per patient. The NMB uses a willingness to pay defined as €5,000 / matched treatment. When the NMB > 0, the graph indicates that for that specific input parameter the benefits outweigh the costs.

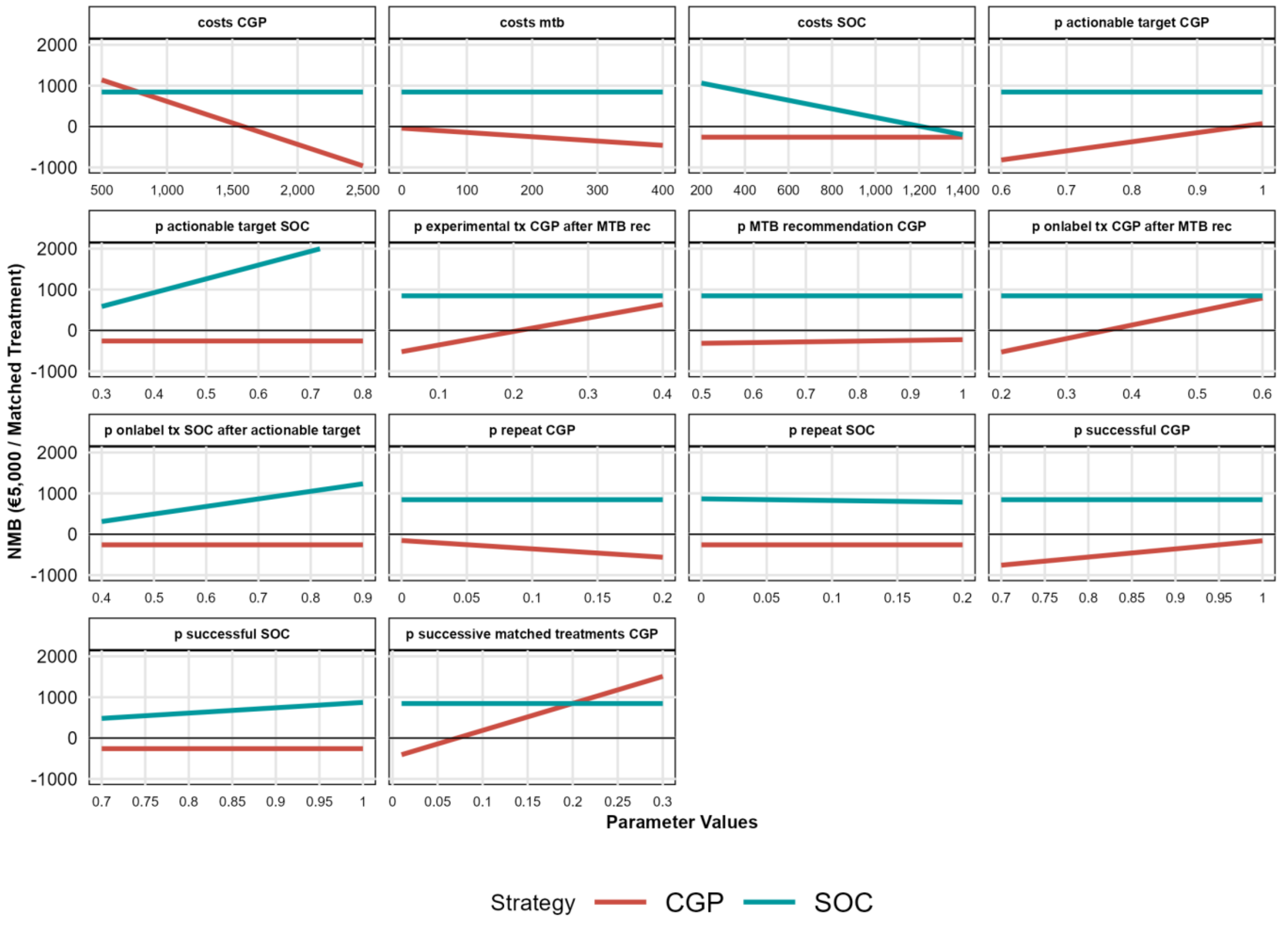

NMB, net monetary benefit, CGP comprehensive genomic profiling, SOC standard of care, MTB molecular tumor board, p probability, tx treatment p probability tx treatment.

© 2025 van Schaik LF et al. *JAMA Network Open*.

eReferences

1. Volders PJ, Aftimos P, Dedeurwaerdere F, Martens G, Canon JL, Beniuga G, et al. A nationwide comprehensive genomic profiling and molecular tumor board platform for patients with advanced cancer. NPJ Precis Oncol. 2025;9(1):66.
2. Maes B. CAN.HEAL WP9 Deliverable report D9.1: National Precision Initiatives. 2025.
3. Vemer P, Corro Ramos I, van Voorn GA, Al MJ, Feenstra TL. AdViSHE: A Validation-Assessment Tool of Health-Economic Models for Decision Makers and Model Users. Pharmacoeconomics. 2016;34(4):349-61.
4. van de Haar J, Hoes LR, Roepman P, Lolkema MP, Verheul HMW, Gelderblom H, et al. Limited evolution of the actionable metastatic cancer genome under therapeutic pressure. Nat Med. 2021;27(9):1553-63.
5. Froyen G, Geerdens E, Berden S, Cruys B, Maes B. Diagnostic Validation of a Comprehensive Targeted Panel for Broad Mutational and Biomarker Analysis in Solid Tumors. Cancers (Basel). 2022;14(10).
6. ComPerMed. Next-generation sequencing guidelines [Available from: https://www.compermed.be/en/guidelines.

© 2025 van Schaik LF et al. *JAMA Network Open*.
