# Supplementary material for: Economic Evaluation of Comprehensive Genomic Profiling in an Advanced Solid Cancer Population
Source: JAMA Netw Open. 2025 Dec 11;8(12):e2548538. doi: 10.1001/jamanetworkopen.2025.48538 (PMC12699362; doi:10.1001/jamanetworkopen.2025.48538)
Supplement: Supplement 2. — Data Sharing Statement [file jamanetwopen-e2548538-s002.pdf]

Data Sharing Statement

van Schaik. Economic Evaluation of Comprehensive Genomic Profiling in an Advanced Solid Cancer Population. *JAMA Netw Open*. Published December 11, 2025.
doi:10.1001/jamanetworkopen.2025.48538

Data
Data available: No

Additional Information
Explanation for why data not available: The clinical datasets utilized as input in this study is available through the original clinical study in the cBioPortal repository, https://www.cbioportal.org/study/summary?id=ballett_bsmo. Volders, PJ., Aftimos, P., Dedeurwaerdere, F. et al. A nationwide comprehensive genomic profiling and molecular tumor board platform for patients with advanced cancer. npj Precis. Onc. 9, 66 (2025). https://doi.org/10.1038/s41698-025-00858-0
